# Proanthocyanidins and Phenolic Compounds from the Twigs of *Salix chaenomeloides* and Their Anti-Lipogenic Effects on 3T3-L1 Preadipocytes

**DOI:** 10.3390/nu16071036

**Published:** 2024-04-02

**Authors:** Kyung Ah Kim, Nguyen Khoi Song Tran, Jiwon Baek, Soah Lee, Ki Sung Kang, Ki Hyun Kim

**Affiliations:** 1 School of Pharmacy, Sungkyunkwan University, Suwon 16419, Republic of Korea; ruddk5480@naver.com (K.A.K.); baekd5nie@gmail.com (J.B.); soahlee@skku.edu (S.L.); 2 College of Korean Medicine, Gachon University, Seongnam 13120, Republic of Korea; kauri87@gachon.ac.kr; 3 Department of Biopharmaceutical Convergence, Sungkyunkwan University, Suwon 16419, Republic of Korea

**Keywords:** *Salix chaenomeloides*, HPLC, NMR, procyanidin B_2_, fatty acid oxidation, anti-lipogenesis

## Abstract

The present study investigated potential bioactive natural products from the EtOH extract of *Salix chaenomeloides* twigs using column chromatography, leading to the isolation of six compounds (**1**–**6**), which were characterized as two proanthocyanidins, procyanidin B_2_ (**1**) and procyanidin B_1_ (**2**), and four phenolic compounds, 4-hydroxybenzoic acid *β*-D-glucosyl ester (**3**), di-*O*-methylcrenatin (**4**), *p*-coumaric acid glucoside (**5**), and syringin (**6**) by the comparison of their NMR spectra with the reported data and high-resolution (HR)-electrospray ionization mass spectroscopy (ESI-MS) analysis. We investigated the potential of six compounds (**1**–**6**) to inhibit adipogenesis in 3T3-L1 preadipocytes, which showed that the compounds (**1**–**6**) significantly reduced lipid accumulation in 3T3-L1 adipocytes without affecting cell proliferation. Notably, compound **1** demonstrated a remarkable 60% and 90% reduction in lipid levels with 50 and 100 µM treatments, respectively. Oil Red O staining results indicated that compound **1** significantly inhibits the formation of lipid droplets, comparable to the effect of T863, an inhibitor of triglyceride used as a positive control, in adipocytes. Compound **1** had no effect on the regulators PPARγ, C/EBPα, and SREBF1 of adipocyte differentiation in 3T3-L1 preadipocytes, but compound **1** activated the fatty acid oxidation regulator, PPARα, compared to the lipogenic-induced control. It also suppressed fatty acid synthesis by downregulating the expression of fatty acid synthase (FAS). Finally, compound **1** induced the mRNA and protein levels of CPT1A, an initial marker of mitochondrial fatty acid oxidation in 3T3-L1. This finding substantiates the anti-lipogenic and lipolytic effects of procyanidin B_2_ (**1**) in 3T3-L1 preadipocytes, emphasizing its pivotal role in modulating obesity-related markers.

## 1. Introduction

Obesity is a multifaceted metabolic disorder characterized by the excessive accumulation of adipose tissue, leading to various modern health complications such as an elevated risk of type 2 diabetes, cardiovascular diseases, and cancer [1,2]. Understanding intracellular lipid metabolism is crucial for researchers seeking insights into improving this prevalent health issue. Adipose tissue serves as the primary site for energy storage, predominantly in the form of triglycerides (TG). It releases stored energy in the form of fatty acids (FA), as needed by the body [3]. In fact, there are key molecular markers that play pivotal roles in regulating lipid metabolism, adipogenesis, and energy homeostasis within adipose tissue [4]. The regulation of adipogenesis involves the coordination of specific transcription factors and regulators inclusive of peroxisome proliferator-activated receptors (PPARs), CCAAT/enhancer-binding protein alpha (C/EBPα), and sterol regulatory element-binding protein 1 (SREBP1), which are crucial regulators of lipid metabolism. PPAR gamma, a member of the PPAR family, is a central regulator of adipocyte differentiation and lipid storage, with documented involvement in adipogenesis and insulin sensitivity [5,6]. Conversely, PPAR alpha is implicated in fatty acid oxidation and energy expenditure, playing a role in modulating hepatic lipid metabolism and mitigating obesity-related complications [7]. C/EBPα, a transcription factor, is vital for adipocyte differentiation and lipid metabolism, regulating the expression of key adipogenic genes to maintain metabolic homeostasis [8]. Lipid homeostasis requires the harmonized expression of the mentioned factors. Another transcription factor, SREBP1, is responsible for synthesizing fatty acids and triglycerides. Overactivation of SREBP1 is linked to increased lipogenesis, contributing to the development of obesity-related complications [9].

Lipid accumulation in the body is predominantly influenced by de novo synthesis of fatty acids. Fatty acid synthase (FAS), an enzyme regulated by insulin, plays a crucial role in de novo lipogenesis. Its upregulation in obesity contributes to excessive fat accumulation and insulin resistance [3,10]. Additionally, adiponectin, an adipokine with insulin-sensitizing and anti-inflammatory properties, also plays an intermediary role in adipogenic differentiation. Reduced levels of adiponectin are associated with obesity-related insulin resistance and cardiovascular complications [11]. Since body energy homeostasis is a key strategy in combating obesity, targeting peroxisomal and mitochondrial β-oxidation, the enzymes Acyl-CoA oxidase (ACOX) and carnitine palmitoyltransferase 1 alpha (CPT1α) have been examined. ACOX, catalyzing the desaturation of acyl-CoA to form 2-trans-enoyl-CoA, is responsible for initiating peroxisomal fatty acid β-oxidation [12]. On the other hand, CPT1α, located at the outer mitochondrial membrane, converts long-chain acyl-CoA to acyl-carnitines, facilitating the transport of fatty acids into mitochondria for breakdown [13].

*Salix chaenomeloides* Kimura, also recognized as *S. glandulosa* Seemen and commonly known as Korean pussy willow in South Korea, is a deciduous shrub and tree belonging to the Salicaceae family. Indigenous to Eastern Asia, including regions of Korea, China, Japan, and Russia, this plant species is known for its ornamental features, ecological significance, and its potential as a source of bioactive compounds with diverse pharmacological properties [14,15]. Members of the genus *Salix* spp. have been revered for millennia as medicinal herbs used to address conditions such as pain, inflammation, and fever. This is attributed to the presence of anti-inflammatory compounds, notably salicylic acid, a widely recognized natural precursor to aspirin, found in their bark [14,15]. In the context of earlier pharmacological investigations of *Salix* spp., a spectrum of therapeutic benefits has been unveiled, including antioxidant [16], anti-tumor [17], anti-inflammatory [18], and anti-obesity effects [19]. Phytochemical studies of the genus *Salix* have reported phenolic compounds, terpenoids, flavonoids, and lignans, which have been linked to diverse biological activities such as cytotoxic, neuroprotective, and antiplasmodial effects [20,21,22,23,24]. Despite these advantageous effects, there is a scarcity of studies on the biological and phytochemical investigations of *S. chaenomeloides*, with only a limited number reported. A prior phytochemical analysis of *S. chaenomeloides* highlighted the presence of flavonoids, phenolic glycosides, and salicin derivatives. Previous pharmacological studies have indicated that these compounds in *S. chaenomeloides* exhibit anti-inflammatory and neuroprotective properties against LPS-induced neuronal death [25,26]. Consequently, further investigations were deemed necessary, which facilitated our exploration of new bioactive compounds from *S. chaenomeloides*.

In our pursuit to explore bioactive natural products from intriguing natural sources [27,28,29,30,31], we embarked on a study to uncover bioactive compounds from the EtOH extract of *S. chaenomeloides* twigs using column chromatography monitored by LC/MS analysis. Here, we describe the isolation and structural elucidation of bioactive compounds from *S. chaenomeloides* twigs, as well as the potential bioactivity of these isolated compounds regarding their anti-lipogenic effects on 3T3-L1 preadipocytes and the possible mechanisms underlying their action.

## 2. Results and Discussion

### 2.1. Isolation and Structural Elucidation of Compounds ***1***–***6***

**Figure 1:**
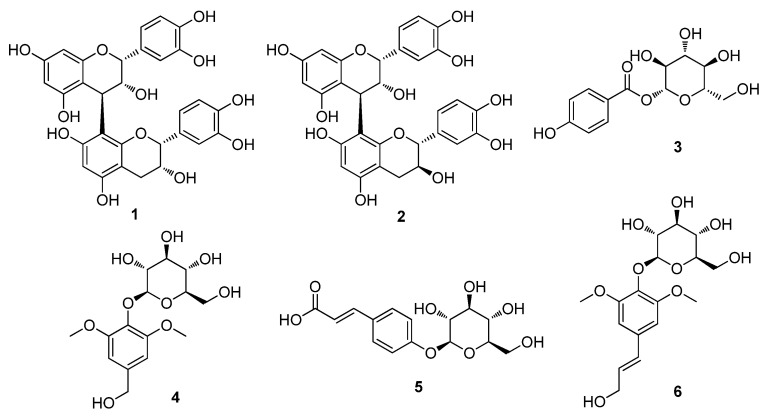
The chemical structures of the isolated compounds **1**–**6**. Procyanidin B_2_ (**1**), procyanidin B_1_ (**2**), 4-hydroxybenzoic acid *β*-D-glucosyl ester (**3**), di-*O*-methylcrenatin (**4**), *p*-coumaric acid glucoside (**5**) and syringin (**6**).

The dried twigs of *S. chaenomeloides* were subjected to extraction with 80% aqueous EtOH. The extract was then processed through a reverse-phase (RP) Sep-Pak column with 100% MeOH to eliminate wax, lipids, and fatty acids. The resulting residue was concentrated using an evaporator, yielding the crude extract. LC/MS analysis of the crude extract identified several peaks corresponding to phenolic compounds through a comparison with our in-house UV library in LC/MS equipment, and these compounds were identified as the main components. Based on these findings, the crude extract underwent phytochemical investigation using continuous column chromatography and semi-preparative HPLC, guided by LC/MS analysis. This extensive analysis led to the isolation of six compounds (**1**–**6**), as illustrated in Figure 1.

The isolated compounds were identified as procyanidin B_2_ (**1**) [32], procyanidin B_1_ (**2**) [32], 4-hydroxybenzoic acid *β*-D-glucosyl ester (**3**) [33], di-*O*-methylcrenatin (**4**) [34], *p*-coumaric acid glucoside (**5**) [35] and syringin (**6**) [36], based on the comparison of their NMR spectra and MS data (Figures S1–S12) with the reported data and high-resolution electrospray ionization mass spectrometry (HR-ESI-MS) analyses. To the best of our knowledge, all of the isolated compounds **1**–**6** were identified for the first time from *S. chaenomeloides*. According to a previous study, syringin, one of the isolated compounds, was found to suppress lipid accumulation in 3T3-L1 cells and significantly reduce the expression of PPAR gamma [37]. Furthermore, a recent study has demonstrated that procyanidin B_2_ inhibits adipogenesis in 3T3-L1 cells, suggesting its potential lipolytic effect on adipose metabolism [38,39]. Nonetheless, the exact mechanism underlying this effect remains incompletely understood. Thus, we examined the inhibitory effects of compounds **1**–**6** on adipogenesis in a murine preadipocyte cell line (3T3-L1).

### 2.2. The Six Compounds (***1***–***6***) Exhibit No Effect on Cell Proliferation While Inhibiting the Lipogenesis of 3T3-L1 Cells

**Figure 2:**
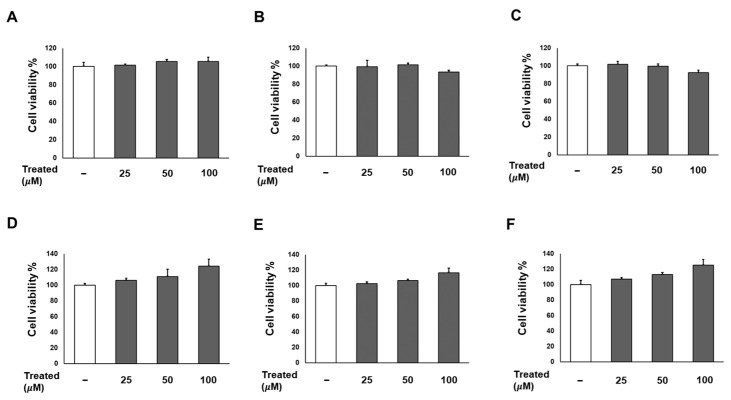
Effects of six compounds (**1**–**6**) on preadipocyte 3T3-L1 proliferation. Cells were seeded in 96-well plates at a density of 1 × 10^4^ cells/well for 24 h. Subsequently, cells were treated with compounds **1** (**A**), **2** (**B**), **3** (**C**), **4** (**D**), **5** (**E**), and **6** (**F**) at concentrations of 25, 50, and 100 µM. Cell viability was determined using an Ez-Cytox cell viability assay kit 24 h after treatment. The data are presented as mean ± standard error of mean (SEM), *n* = 5, analyzed using *t*-test statistics.

Firstly, we conducted a screening for the cytotoxicity of six compounds (**1**–**6**) in 3T3-L1 preadipocytes in a dose-dependent manner (0, 25, 50, and 100 µM). After a 24-h incubation, the cell proliferation percentages for all six compounds at each treatment dose showed no significant difference compared to the non-treated group (Figure 2). Consequently, the treatments with these compounds were deemed safe for adipose tissue.

**Figure 3:**
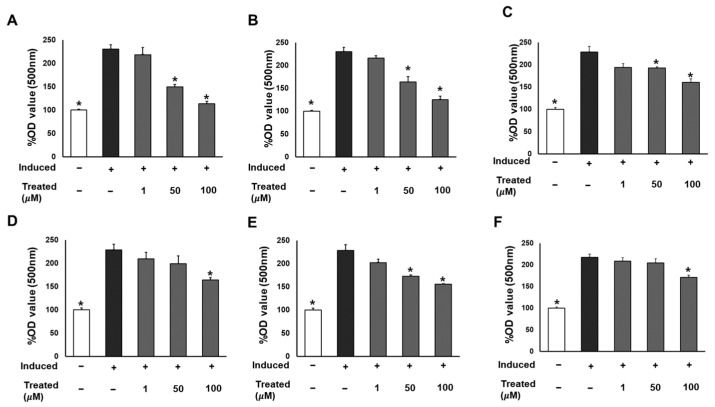
Inhibitory effects of six compounds (**1**–**6**) on lipogenesis in 3T3-L1 preadipocytes. Cells were seeded in 48-well plates at a density of 5 × 10^4^ cells/well for 24 h and treated with compounds **1** (**A**), **2** (**B**), **3** (**C**), **4** (**D**), **5** (**E**), and **6** (**F**) (1, 50, and 100 µM) during the induction of 3T3-L1 preadipocyte differentiation. Cell lipid droplets were determined using Oil Red O staining after 7 days of differentiation, and staining efficiency was evaluated by OD value at 500 nm wavelength. Data are presented as mean ± standard error of mean (SEM), *n* = 4 using *t*-test statistic. (*) *p* < 0.05 versus induced only.

To assess the effect of the six compounds (**1**–**6**) on 3T3-L1 lipogenic differentiation, 3T3-L1 cells were exposed to the compounds at various concentrations (0, 1, 50, and 100 µM) during the induction of differentiation. After 7 days of induction, cells were stained with Oil Red O for lipid droplet determination (Figure 3). The results indicated a significant reduction in lipid accumulation levels in adipocytes exposed to the six compounds at 100 µM compared to the induced cells. Particularly noteworthy was a 60% and 90% reduction in lipid levels with 50 and 100 µM treatment of compound **1**, respectively. The observed alleviation of lipid droplets and fat accumulation suggests the inhibitory effect of the six compounds, especially compound **1**, on lipogenic differentiation.

### 2.3. Compound ***1*** Has No Effect on 3T3-L1 Cell Proliferation in a Time- and Dose-Dependent Manner

**Figure 4:**
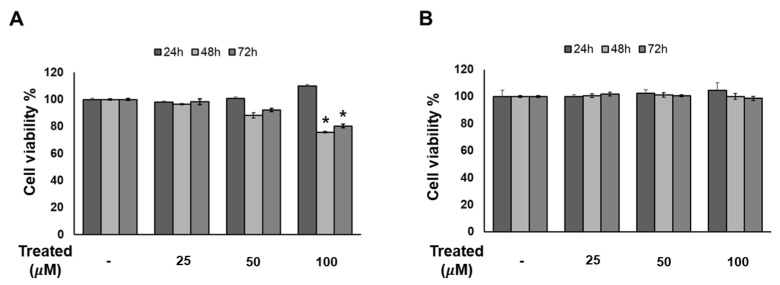
Effect of compound **1** on the proliferation of 3T3-L1 cells. The cells were seeded in 96-well plates at a density of 1 × 10^4^ cells/well for 24 h. Cells were treated with T863 (**A**) and **1** (**B**) at concentrations of 25, 50, and 100 µM. Cell viability was determined using an Ez-Cytox cell viability assay kit for 24, 48, and 72 h after treatment. Data are presented as mean ± standard error of mean (SEM), *n* = 5 using *t*-test statistic. (*) *p* < 0.05 versus non-treated at a similar time point.

Since compound **1** demonstrated the strongest inhibition of adipogenesis among the screening compound set, we initially conducted a time- and dose-dependent cytotoxicity test of compound **1** on 3T3-L1 preadipocytes (Figure 4). Cells were exposed to compound **1** at various doses (0, 25, 50, and 100 µM) for three time periods: 24, 48, and 72 h. After 72 h of treatment, no toxicity was observed at any concentration. Additionally, T863, inhibitor of triglyceride, which is a major component of lipid droplet formation, was used as a positive control. After 48 and 72 h of exposure to T863, the cell proliferation rate was reduced by 20% with a concentration of 100 µM (Figure 4). Therefore, long-term exposure to lipogenesis inhibitors might cause a decrease in cell viability, but compound **1** was deemed safe for 3T3-L1 cells.

### 2.4. Compound ***1*** Significantly Inhibits the Formation of Lipid Droplets in Adipocytes

**Figure 5:**
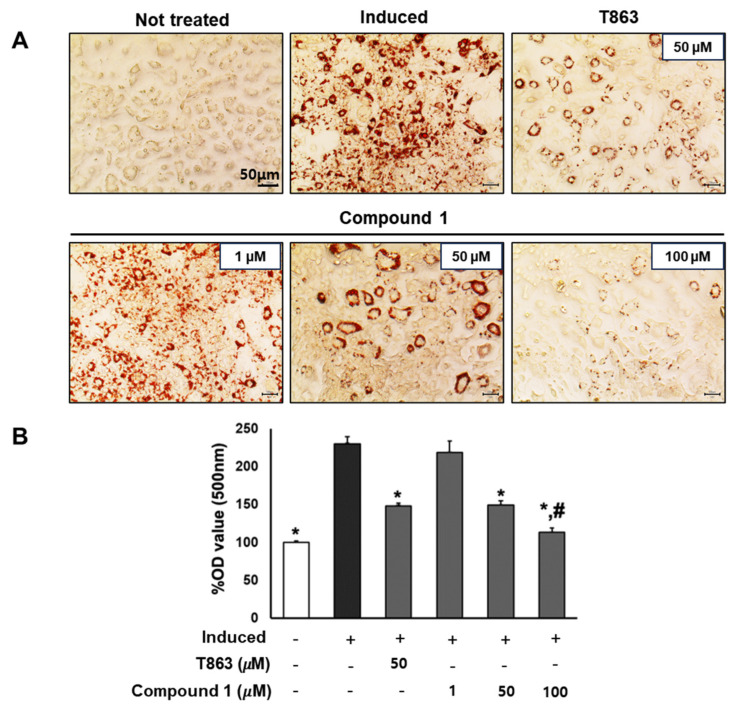
Inhibitory effect of compound **1** on the lipogenesis of preadipocyte 3T3-L1. The cells were seeded in 48-well plates at a density of 5 × 10^4^ cells/well for 24 h and treated with T863 (50 µM) and compound **1** (1, 50, and 100 µM) while inducing 3T3-L1 preadipocyte differentiation. Cell lipid droplets were determined using Oil Red O staining after 7 days of differentiation and treatments (**A**). Staining efficiency was evaluated by OD value at 500 nm wavelength (**B**). Data are presented as mean ± standard error of mean (SEM), *n* = 4 using *t*-test statistic. (*) *p* < 0.05 versus induced only group. (#) *p* < 0.05 versus T863-treated group.

To confirm the lipogenesis inhibition effects of compound **1**, 3T3-L1 cells underwent lipogenic differentiation induction and were exposed to T863 (50 µM) and compound **1** (0, 1, 50, and 100 µM) over a 7-day period. Oil Red O results on day 7 depicted the lipid accumulation for each treatment group (Figure 5). Significantly, a reduction of over 60% in lipid levels was observed in samples treated with 50 µM of compound **1**, comparable to the effect of T863. Furthermore, compound **1** demonstrated a remarkable inhibition of lipid formation, by 90% at the 100 µM dose, which was significantly lower than in the T863-treated group (Figure 5). These findings highlight the potential of compound **1** in effectively modulating lipid accumulation during the differentiation process.

### 2.5. Compound ***1*** Has No Effect on Regulators of Adipocyte Differentiation in 3T3-L1 Preadipocytes but Activates Fatty Acid Oxidation Regulator

**Figure 6:**
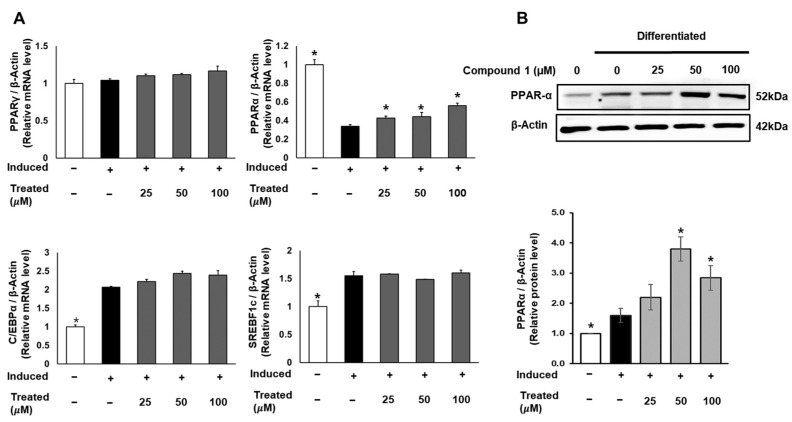
Effect of compound 1 on regulators of adipocyte differentiation in 3T3-L1 preadipocytes. Cells were seeded in 6-well plates at a density of 5 × 10^5^ cells/well for 24 h and treated with compound **1** (25, 50, and 100 µM) while inducing lipogenic differentiation. After 24 h of treatment, mRNA expression of PPARγ, PPARα, C/EBPα, and SREBF1c was determined using qPCR (**A**). The protein expression of PPARα was measured by Western blotting, and relative expression levels were quantified using the ImageJ program (**B**). Data are presented as mean ± standard error of mean (SEM), *n* = 3, analyzed using the *t*-test statistic. (*) *p* < 0.05 versus induced-only group.

To uncover the molecular mechanism of the anti-lipogenic activity of compound **1**, we conducted mRNA and protein expression tests. 3T3-L1 cells were induced for lipogenic differentiation and treated with compound **1** (0, 25, 50, 100 µM) for 24 h. The mRNA expression levels of PPARγ, PPARα, C/EBPα, and SREBF1 in both control and treated groups were examined using qPCR and normalized to the β-actin gene (Figure 6). Notably, compound **1** demonstrated no dose-dependent effect on the expression of PPARγ, C/EBPα, and SREBF1. However, compound **1** dose-dependently upregulated the mRNA levels of PPARα compared to the lipogenic-induced control. Subsequently, the protein expression of PPARα was examined and normalized to β-actin protein (Figure 6). At doses of 50 and 100 µM, compound **1** significantly improved the expression of PPARα protein, indicating the activation of fatty acid metabolism and the inhibition of triglyceride formation.

### 2.6. Compound ***1*** Suppresses Fatty Acid Synthesis but Has No Influence on Insulin-Sensitive Marker in 3T3-L1 Cells

**Figure 7:**
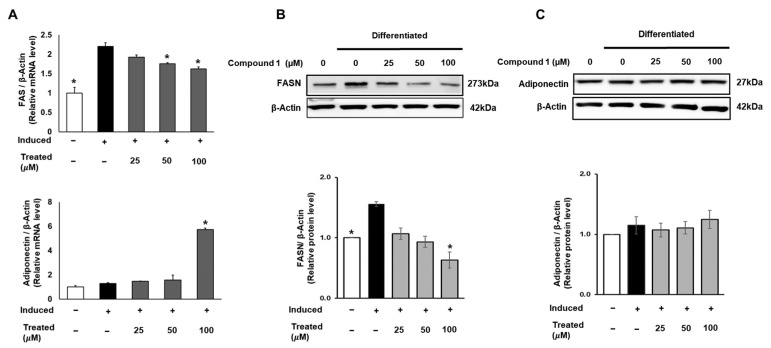
Effect of compound **1** on de novo lipogenesis and insulin sensitivity in 3T3-L1 preadipocytes. Cells were seeded in 6-well plates at a density of 5 × 10^5^ cells/well for 24 h and treated with compound **1** (25, 50, and 100 µM) while inducing lipogenic differentiation. After 24 h of treatment, mRNA expression of FAS and adiponectin was determined using qPCR (**A**). Protein expression of FAS (**B**) and adiponectin (**C**) was measured by Western blotting, and relative expression levels were quantified using the ImageJ program. Data are presented as mean ± standard error of mean (SEM), *n* = 3, analyzed using the *t*-test statistic. (*) *p* < 0.05 versus induced-only group.

FAS is a crucial enzyme that regulates the synthesis of long-chain fatty acids, which are subsequently stored in fat cells. The accumulation of fatty acids is closely associated with insulin sensitivity in adipocytes [3,10]. Therefore, in this study, we targeted FAS and the insulin-sensitizing marker adiponectin. To assess the expression of FAS and adiponectin, 3T3-L1 cells were treated with compound **1** (0, 25, 50, 100 µM) during lipogenic differentiation for 24 h. The mRNA levels of FAS and adiponectin were examined using qPCR and normalized to β-actin (Figure 7). Compound **1** exhibited a suppressive effect on the mRNA expression of FAS in a dose-dependent manner. At a treatment of 100 µM of compound **1**, the mRNA expression of FAS was reduced by 26% compared to that of the induced group. Concurrently, a 6-fold elevation in adiponectin mRNA levels was observed at the same dose of treatment (Figure 7). In addition, to assess the total protein expression of these two genes, we conducted Western blotting for FASN and adiponectin. A similar pattern for compound **1** was observed with the FASN protein level, showing a decline of up to 60% in total FAS expression compared to the induced control (Figure 7). Although there was a significant increase in adiponectin mRNA levels, no change was found in the total protein expression (Figure 7).

### 2.7. Compound ***1*** Induces Mitochondrial Fatty Acid Oxidation in 3T3-L1

**Figure 8:**
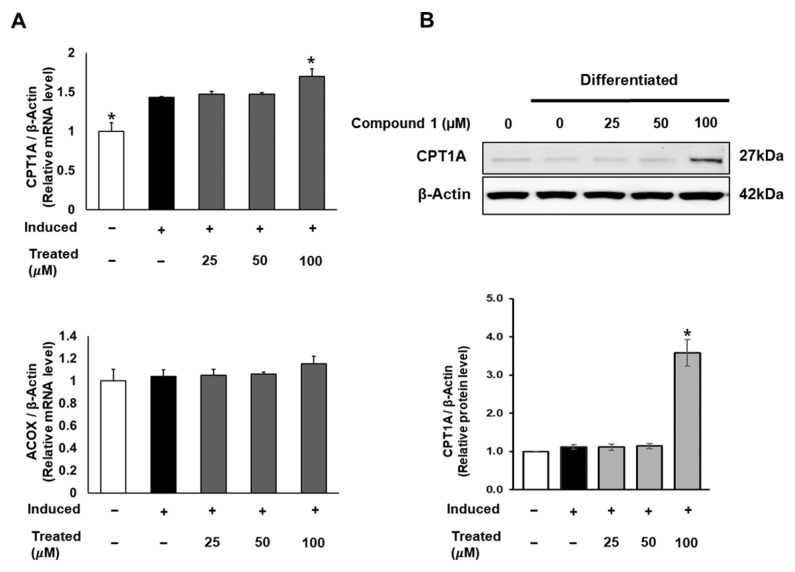
Effect of compound **1** on mitochondrial fatty acid oxidation in 3T3-L1 cells. Cells were seeded in 6-well plates at a density of 5 × 10^5^ cells/well for 24 h and treated with compound **1** (25, 50, and 100 µM) during lipogenic differentiation. After 24 h of treatment, mRNA expression of CPT1A and ACOX was determined using qPCR (**A**). Protein expression of CPT1A (**B**) was measured by Western blotting, and relative expression levels were quantified using the ImageJ program. Data are presented as mean ± standard error of mean (SEM), *n* = 3, analyzed using the *t*-test statistic. (*) *p* < 0.05 versus induced-only group.

In addition to lipogenesis and fatty acid storage, fatty acid β-oxidation plays a pivotal role in lipid metabolism and obesity studies [13]. To explore the potential effects of compound **1** on the fatty acid oxidation process, we investigated the mRNA and protein expression of CPT1 and ACOX, which serve as initial markers of β-oxidation in mitochondria and peroxisomes [38]. While compound **1** showed no effect on ACOX mRNA expression, it notably increased the mRNA and protein levels of CPT1A with a 100 µM dose treatment (Figure 8).

**Figure 9:**
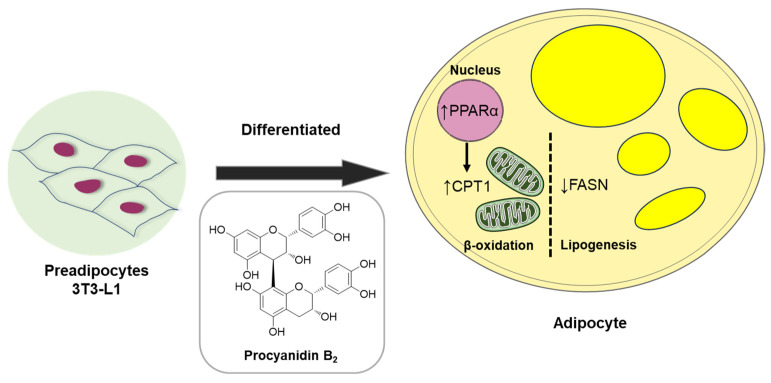
Schematic illustration depicting the underlying mechanism of the effect of compound **1** (procyanidin B_2_) on metabolism in differentiated 3T3-L1 cells.

Collectively, our data demonstrate the anti-lipogenic and lipolytic effects of compound **1** (procyanidin B_2_) in vitro in 3T3-L1 preadipocytes. These effects were associated with the fatty acid oxidation regulator, PPARα, and markers such as fatty acid synthase (FAS) and CPT1A, an initial marker of mitochondrial fatty acid oxidation in 3T3-L1 adipocyte differentiation (Figure 9). Considerable evidence suggests that lipid accumulation or storage during the differentiation of preadipocytes into adipocytes involves the upregulation of enzymes like FAS [39]. FAS, highly expressed in various tissues including adipose tissue, plays a key role in catalyzing fatty acid synthesis [40]. Intriguingly, our study illustrated that procyanidin B_2_ significantly down-regulates mRNA and protein expression levels of FAS and CPT1A in differentiating 3T3-L1 cells.

Procyanidins have been associated with a lipolytic effect on adipose metabolism [39], but the underlying mechanism remains incompletely understood. This study, therefore, sheds light on the anti-lipogenic and lipolytic effects and the underlying mechanisms of one specific procyanidin, procyanidin B_2_, during adipocyte differentiation. Although further studies are needed, procyanidin B_2_ may hold promise in the development of therapies for obesity-related metabolic disorders. The role of obesity and related markers underscores the intricate molecular network governing lipid metabolism and adipose tissue function. This research significantly contributes to our understanding of these markers and their implications in obesity-related complications.

Therapeutic strategies for obesity typically target both fat accumulation (lipogenesis) and fat breakdown (lipolysis) in the body. Compound **1** (procyanidin B_2_), an anti-lipogenic agent identified in this study, inhibited fat synthesis and storage, thereby reducing adipose tissue buildup. Additionally, it promoted the breakdown of stored fat for energy expenditure. Combining these approaches can enhance weight management and improve obesity treatment outcomes by targeting specific lipid metabolism enzymes or pathways. For example, inhibitors of key enzymes like FAS or acetyl-CoA carboxylase (ACC) can reduce fatty acid and triglyceride production, thereby limiting fat accumulation. Conversely, activators of lipolytic enzymes such as hormone-sensitive lipase (HSL) or adipose triglyceride lipase (ATGL) can facilitate fat breakdown. Another approach can involve modulating hormone signaling pathways that regulate lipid metabolism and energy balance. Hormones like leptin, adiponectin, insulin, and glucagon play crucial roles in appetite control, fat storage, and energy expenditure. Compounds that mimic these hormones or regulate their secretion and activity can influence lipid metabolism and aid in managing obesity. Lifestyle interventions, including dietary adjustments and regular exercise, can complement pharmacological treatments. A balanced diet that restricts excess calories and saturated fats while emphasizing nutrient-rich foods supports weight loss efforts. Consistent physical activity not only increases energy expenditure but also enhances lipid mobilization and metabolism.

Indeed, obesity is frequently associated with various comorbidities, including type 2 diabetes, cardiovascular disease, and metabolic syndrome. To effectively address the complexities of obesity and its related health complications, future research efforts should focus on developing holistic and multidisciplinary approaches to obesity management. These approaches should aim to not only promote weight loss but also prevent and manage obesity-related complications. One potential strategy can involve integrating pharmacological, behavioral, and surgical interventions within a coordinated care framework. By combining these different modalities, healthcare providers can offer comprehensive treatment plans tailored to individual patient needs. In addition to these interventions, a multidisciplinary approach to obesity management should involve collaboration between healthcare professionals from various disciplines, including physicians, dietitians, psychologists, and exercise physiologists. This team-based approach ensures that patients receive comprehensive care that addresses their medical, nutritional, psychological, and physical needs.

The findings of this study hold significant translational implications, providing valuable insights into the intricate landscape of obesity-related complications. By uncovering the mechanisms through which natural products can alleviate obesity-related health risks, we have enhanced our understanding and potential for developing novel treatments for diseases associated with obesity. Moreover, the translational impact of these findings extends beyond the laboratory, paving the way for innovative therapeutic strategies and personalized treatment approaches for individuals grappling with obesity and its related complications. Through deepening our comprehension of obesity at the molecular level, this research can contribute to broader efforts aimed at combating the global epidemic of obesity and fostering improved public health outcomes.

## 3. Materials and Methods

### 3.1. General Experimental Procedures

The equipment and devices used in the analyses and experiments are listed in Table 1.

### 3.2. Plant Material

In June 2021, mature twigs were collected from *S. chaenomeloides* growing in a marsh in Gyeongju-si, Gyeongsangbuk-do, Republic of Korea, at an altitude of 60 m. The geographic coordinates of the collection site were recorded as 35°58′26.9″ N, 129°09′15.5″ E. The authentication process was conducted by J.N. Yu, the author. Voucher specimens (HIMH-2105) have been securely stored at the Hongcheon Institute of Medicinal Herbs in Hongcheon-gun, Republic of Korea.

### 3.3. Extraction and Isolation

Twigs of *S. chaenomeloides* (6.7 kg) were dried at 40 °C in a grain dryer for 72 h and pulverized. The dried leaves (1.2 kg) were subjected to extraction with 80% ethanol (10 L) using sonication for 90 min, repeated three times at room temperature. The filtered ethanol extract was evaporated *in vacuo*, yielding the crude ethanol extract (111.4 g, extraction yield = 9.29%). Subsequently, 20.8 g of the crude extract was dissolved in MeOH (100 mL) and applied to an RP Sep-Pak column with 100% MeOH to eliminate wax, lipids, and fatty acids. The resulting residue was concentrated using an evaporator to obtain the crude extract (12.2 g). The crude extract (7.1 g) underwent preparative RP-HPLC (from 20 to 80% MeOH for 80 min, gradient system) to produce three fractions (A–C). The B fraction (2.2 g) was subjected to silica open column chromatography with a gradient solvent system of CH_2_Cl_2_/MeOH (15:1, CH_2_Cl_2_/MeOH to 100% MeOH), resulting in four fractions (B1–B4). The B2 fraction (633 mg) was further purified by preparative RP-HPLC (from 25 to 75% MeOH/H_2_O for 80 min, gradient system) to yield five fractions (B21–B25). Subfraction B22 (80.1 mg) underwent semi-preparative reversed-phase HPLC with a 22% MeOH/H_2_O isocratic solvent system, leading to the isolation of compounds **1** (1.8 mg, *t_R_* = 48.1 min) and **2** (2.4 mg, *t_R_* = 60.2 min). Similarly, subfraction B23 (57.0 mg) was subjected to semi-preparative reversed-phase HPLC with a 14% MeOH/H_2_O isocratic solvent system to isolate compounds **3** (2.2 mg, *t_R_* = 29.3 min) and **4** (2.4 mg, *t_R_* = 33.2 min). Subfraction B24 (67.2 mg) underwent semi-preparative reversed-phase HPLC with a 29% MeOH/H_2_O isocratic solvent system, resulting in the isolation of compound **5** (1.4 mg, *t_R_* = 22.7 min). Fraction B3 (697 mg) was subjected to preparative RP-HPLC (from 25 to 70% MeOH/H_2_O for 80 min, gradient system) to obtain five fractions (B31–B34). Subfraction B31 (55.5 mg) underwent semi-preparative reversed-phase HPLC with a 24% MeOH/H_2_O isocratic solvent system, leading to the isolation of compound **6** (2.2 mg, *t_R_* = 47.3 min).

### 3.4. Cell Culture

The culturing of murine 3T3-L1 preadipocytes involved the use of Dulbecco’s modified Eagle’s medium (Corning, Manassas, VA, USA), supplemented with 10% Bovine Calf Serum (Thermo Scientific, Waltham, MA, USA), and 100 units/mL penicillin with 100 mg/mL streptomycin (Gibco, NY, USA). The cells with passage number between 10–20 were incubated under standard conditions at 37 °C in a humidified atmosphere with 5% CO_2_. Sub-culturing was performed when cells reached 90% confluence, facilitating the preparation of cells for specific assays.

### 3.5. Cell Viability Assay

3T3-L1 cells were initially seeded in a 96-well plate at a density of 1 × 10^4^ cells/well and allowed to incubate for 24 h. Subsequently, the cells underwent treatment with six isolated compounds (**1**–**6**) across varying concentrations (0, 25, 50, and 100 µM) to assess cell proliferation rates. Following 24, 48, and 72 h treatment periods, cell viability was determined using the EZ-Cytox assay reagent (DoGen, Seoul, Republic of Korea) for each well. Absorbance at 450 nm was then measured using a microplate reader (PowerWave XS; Bio-Tek Instruments, Winooski, VT, USA) to quantify cell viability.

### 3.6. Oil Red O Assay

Preadipocytes were sub-cultured in 48-well plates at a density of 5 × 10^4^ cells/well for 24 h and treated with T863 (50 µM) and six compounds (0, 1, 50, and 100 µM) while inducing lipogenic differentiation. Dulbecco’s modification of Eagle’s medium (Corning, Manassas, VA, USA) supplemented with 10% Fetal Bovine Serum (Atlas Biologicals, Fort Collins, CO, USA), 100 units/mL penicillin, 100 mg/mL streptomycin (Gibco, Grand Island, NY, USA), and insulin solution (Sigma-Aldrich, St. Louis, MO, USA) was used for differentiation. After 7 days of differentiation and treatments, cells were fixed and washed with DPBS. Cell lipid droplets were observed using Oil Red O staining (Sigma-Aldrich, St. Louis, MO, USA). Staining efficiency was evaluated by the OD value at 500 nm wavelength to determine the lipid accumulation level in differentiated and treated groups.

### 3.7. mRNA Expression Measurement by Quantitative qPCR

Preadipocyte 3T3-L1 cells were cultured in a 6-well plate at a density of 5 × 10^5^ cells/well one day prior and subsequently treated with compound **1** at specified concentrations (0, 25, 50, and 100 µM) for a 24-h duration. Following treatment, cells were harvested for mRNA extraction using Trizol (Thermo Scientific, Waltham, MA, USA). The quantification of mRNA expression was carried out through the quantitative PCR method, utilizing AccuPower^®^ 2X GreenStar™ qPCR Master Mix (Bioneer, Deajeon, Republic of Korea). This approach allowed for the assessment of gene expression in response to the various compound concentrations.

### 3.8. Western Blotting Analysis for Protein Expression

3T3-L1 cells were seeded in a 6-well plate at a density of 1 × 10^6^ cells/well for 24 h and treated with compound **1** at indicated concentrations (0, 25, 50, 100 µM). After 24 h, cells were collected and lysed in RIPA buffer (Tech&Innovation, Gangwon, Republic of Korea) following the manufacturer’s instructions to obtain whole-cell extracts. Protein concentration was determined using the Pierce™ BCA Protein Assay Kit (Thermo Scientific, Waltham, MA, USA). The proteins separated by electrophoresis were transferred onto PVDF membranes. Primary antibodies, including PPARα, FAS, adiponectin, CPT1A, and β-actin, were used in combination with conjugated secondary antibodies (Cell Signaling, Boston, MA, USA) to label the target proteins. The bound antibodies were detected using Pierce™ ECL Advance Western Blotting Detection Reagents (Thermo Scientific, Waltham, MA, USA) and visualized with the FUSION Solo Chemiluminescence System (PEQLAB Biotechnologie GmbH, Erlangen, Germany).

### 3.9. Statistics Analysis

The data were expressed as mean ± standard error of mean (SEM) and analyzed using the student t-test statistic (GraphPad Prism, San Diego, CA, USA). *p* values less than 0.05 versus control groups were considered statistically significant.

## 4. Conclusions

In this study, we embarked on a phytochemical exploration of the EtOH extract derived from the twigs of *S. chaenomeloides*, a traditional herbal medicine. This investigation resulted in the isolation and identification of two proanthocyanidins, procyanidin B_2_ (**1**) and procyanidin B_1_ (**2**), and four phenolic compounds, 4-hydroxybenzoic acid *β*-D-glucosyl ester (**3**), di-*O*-methylcrenatin (**4**), *p*-coumaric acid glucoside (**5**), and syringin (**6**), guided by LC/MS analysis. The chemical structures of the isolates (**1**–**6**) were elucidated through NMR techniques and HR-ESIMS. All of the isolated compounds **1**–**6** were identified for the first time from *S. chaenomeloides*. During the study investigating the effects of six compounds (**1**–**6**) on adipogenesis in 3T3-L1 preadipocytes, compound **1** demonstrated a significant reduction in lipid accumulation levels and inhibited the formation of lipid droplets, showcasing its anti-lipogenic and lipolytic effects. Additionally, compound **1** (procyanidin B_2_) activated fatty acid oxidation regulators, PPARα and CPT1A, and suppressed fatty acid synthesis by downregulating fatty acid synthase (FAS) expression, emphasizing its potential role in modulating obesity-related markers in 3T3-L1 preadipocytes. By exploring the molecular mechanisms underlying these effects, the current study advances our understanding of the intricate interplay between natural products and physiological processes related to obesity. This also gives rise to the hypothesis that procyanidin B_2_ is a potential candidate for an anti-obesity therapeutic approach. However, the molecular mechanism of action of this compound on the target individuals can be further studied using preclinical experimental settings in the near future.

## Figures and Tables

**Table 1:** Equipment and devices used for analyses.

Experimental Procedure	Equipment
Ultraviolet (UV) spectra	Agilent 8453 UV-visible spectrophotometer (Agilent Technologies, Santa Clara, CA, USA)
Nuclear magnetic resonance (NMR) spectra	Bruker AVANCE III HD 850 NMR spectrometer with a 5 mm TCI CryoProbe operating at 850 MHz (^1^H) and 212.5 MHz (^13^C)
HR-ESIMS	Agilent G6545B quadrupole time-of-flight mass spectrometer (Agilent Technologies) Agilent 1290 Infinity II high-performance liquid chromatography (HPLC) instrument [Agilent Eclipse Plus C18 column (2.1 × 50 mm, 1.8 μm; flow rate: 0.3 mL/min)]
Preparative HPLC	Waters 1525 Binary HPLC pump with a Waters 996 Photodiode Array Detector (Waters Corporation, Milford, MA, USA) and an Agilent Eclipse C18 column (250 × 21.2 mm, 5 μm; flow rate: 5 mL/min; Agilent Technologies)
Semi-preparative HPLC	Waters 1525 Binary HPLC pump with a Waters 996 Photodiode Array Detector (Waters Corporation, Milford, CT, USA) Phenomenex Luna C18 column (250 × 10 mm, 10 μm; flow rate: 2 mL/min; Phenomenex) Phenomenex Luna Phenyl-Hexyl column (250 × 10 mm, 10 μm; flow rate: 2 mL/min; Phenomenex)
LC/MS analysis	Agilent 1200 Series HPLC system (Agilent Technologies, Santa Clara, CA, USA) equipped with a diode array detector and 6130 Series electrospray ionization (ESI) mass spectrometer (Agilent Technologies) using an analytical Kinetex C18 100 Å column (100 × 2.1 mm, 5 μm; flow rate: 0.3 mL/min; Phenomenex) set at 35 °C. ESI conditions: capillary voltage, 2.0 kV; convoltage, 50 V; source temperature, 120 °C; desolvation temperature, 350 °C; desolvation gas flow rate, 800 L/h. Nebulizer and auxiliary gas: high-purity nitrogen. Injecting column: 10 μL. The mobile phase: formic acid in H_2_O (0.1% [*v*/*v*]) (A) and methanol (B). Chromatographic conditions: the programmed gradient elution of 10%–100% (B) for 30 min, 100% (B) for 1 min, 100% (B) isocratic for 10 min, and then 10% (B) isocratic for 10 min to perform post-run reconditioning of the column.
Column chromatography	Silica gel 60 (230-240 mesh; Merck, Darmstadt, Germany) Sep-Pak C18-E (55 μm, 70Å, 2 g/12 mL giga tubes, Phenomenex)
Thin-layer chromatography (TLC)	Pre-coated silica gel F254 plates and RP-C18 F254s plates (Merck)

## Data Availability

Data are contained within the article and Supplementary Materials.

## Supplementary Materials

The following supporting information can be downloaded at: https://www.mdpi.com/article/10.3390/nu16071036/s1, Figure S1: The LC-UV chromatogram at 210 nm, UV spectrum and negative-ion mode MS data of **1**; Figure S2: The ^1^H NMR spectrum of **1** (CD_3_OD, 850 MHz); Figure S3: The LC-UV chromatogram at 210 nm, UV spectrum and negative-ion mode MS data of **2**; Figure S4: The ^1^H NMR spectrum of **2** (CD_3_OD, 850 MHz); Figure S5: The LC-UV chromatogram at 254 nm, UV spectrum and negative-ion mode MS data of **3**; Figure S6: The ^1^H NMR spectrum of **3** (DMSO, 850 MHz); Figure S7: The LC-UV chromatogram at 210 nm, UV spectrum and negative-ion mode MS data of **4**; Figure S8: The ^1^H NMR spectrum of **4** (DMSO, 850 MHz); Figure S9: The LC-UV chromatogram at 210 nm, UV spectrum and negative-ion mode MS data of **5**; Figure S10: The ^1^H NMR spectrum of **5** (CD_3_OD, 850 MHz); Figure S11: The LC-UV chromatogram at 210 nm, UV spectrum and negative-ion mode MS data of **6**; Figure S12: The ^1^H NMR spectrum of **6** (CD_3_OD, 850 MHz).
