# Supplementary material for: Proanthocyanidins and Phenolic Compounds from the Twigs of Salix chaenomeloides and Their Anti-Lipogenic Effects on 3T3-L1 Preadipocytes
Source: Nutrients. 2024 Apr 2;16(7):1036. doi: 10.3390/nu16071036 (PMC11013749; doi:10.3390/nu16071036)

# Supplementary Materials

## Supporting Information Contents:

**Figure S1.** The LC-UV chromatogram at 210 nm, UV spectrum and negative-ion mode MS data of **1**……S3
**Figure S2.** The $^1$H NMR spectrum of **1** ($CD_3OD$, 850 MHz) ……S4
**Figure S3.** The LC-UV chromatogram at 210 nm, UV spectrum and negative-ion mode MS data of **2**……S5
**Figure S4.** The $^1$H NMR spectrum of **2** ($CD_3OD$, 850 MHz) ……S6
**Figure S5.** The LC-UV chromatogram at 254 nm, UV spectrum and negative-ion mode MS data of **3**……S7
**Figure S6.** The $^1$H NMR spectrum of **3** (DMSO, 850 MHz) ……S8
**Figure S7.** The LC-UV chromatogram at 210 nm, UV spectrum and negative-ion mode MS data of **4**……S9
**Figure S8.** The $^1$H NMR spectrum of **4** (DMSO, 850 MHz) ……S10
**Figure S9.** The LC-UV chromatogram at 210 nm, UV spectrum and negative-ion mode MS data of **5**……S11
**Figure S10.** The $^1$H NMR spectrum of **5** ($CD_3OD$, 850 MHz) ……S12
**Figure S11.** The LC-UV chromatogram at 210 nm, UV spectrum and negative-ion mode MS data of **6**……S13
**Figure S12.** The $^1$H NMR spectrum of **6** ($CD_3OD$, 850 MHz) ……S14

**Figure S1.** The LC-UV chromatogram at 210 nm, UV spectrum and negative-ion mode MS data of **1**

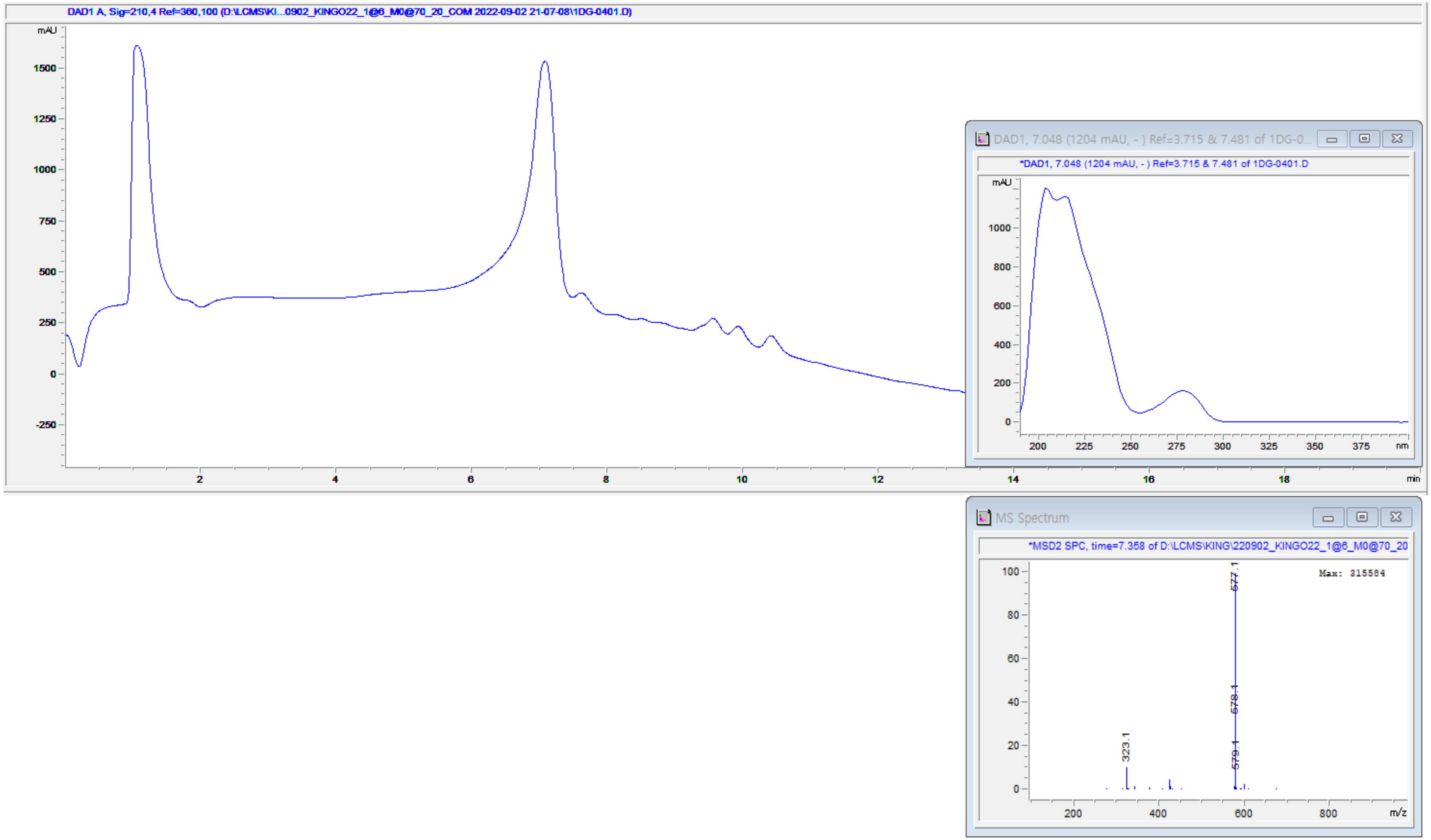

**Figure S2.** The $^{1}$H NMR spectrum of **1** ($CD_3OD$, 850 MHz)

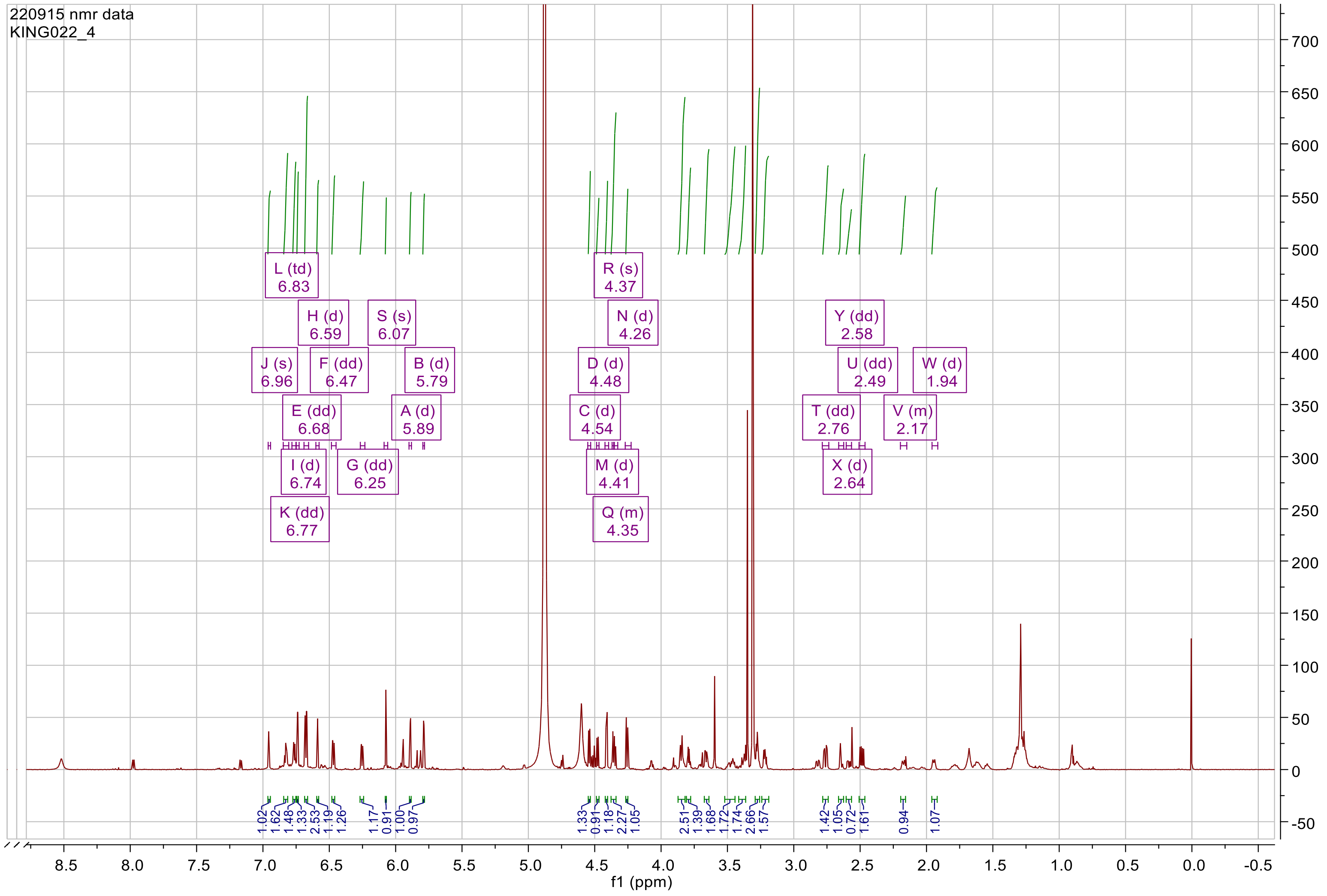

**Figure S3.** The LC-UV chromatogram at 210 nm, UV spectrum and negative-ion mode MS data of **2**

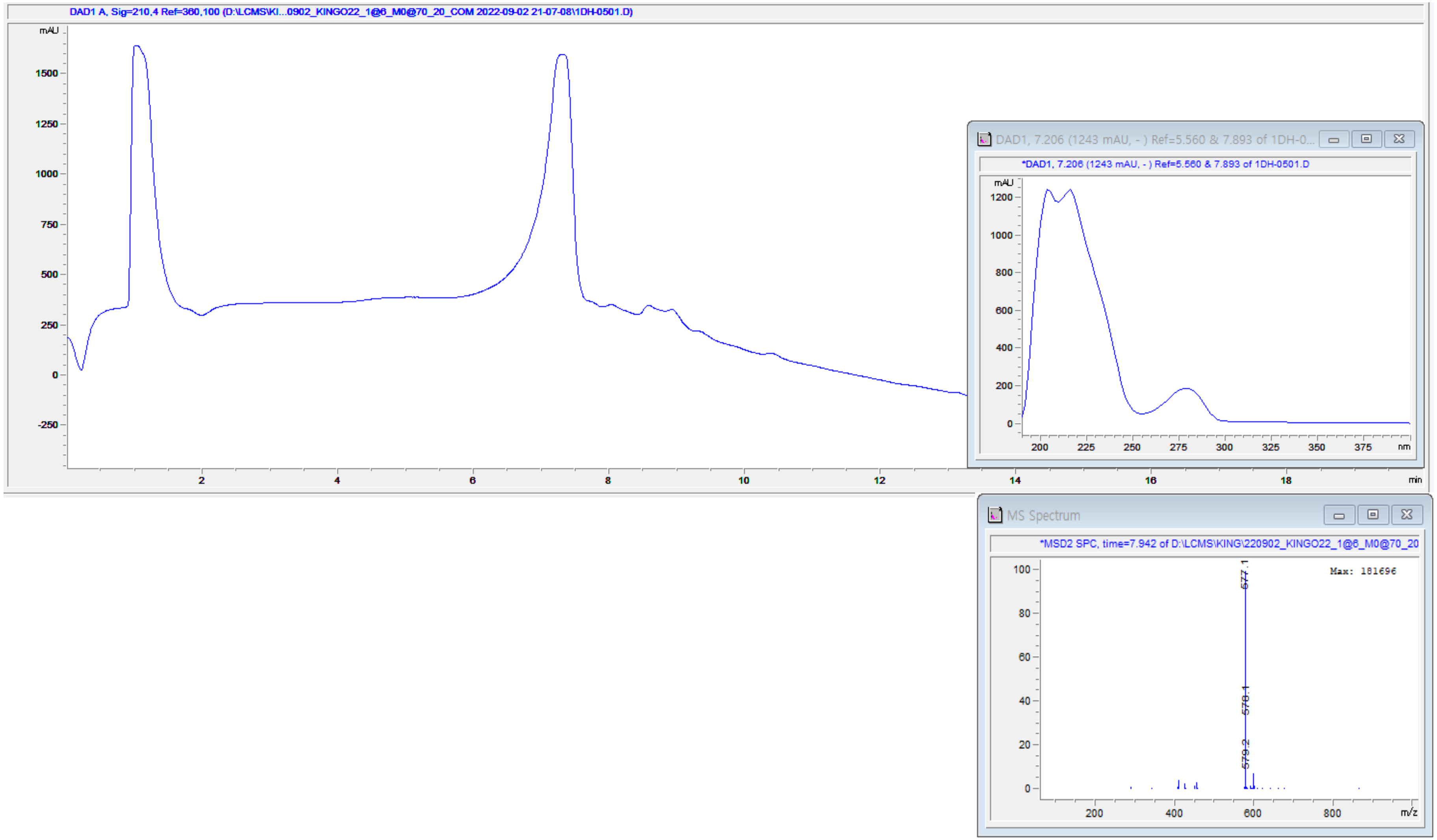

**Figure S4.** The $^{1}$H NMR spectrum of **2** ($CD_3OD$, 850 MHz)

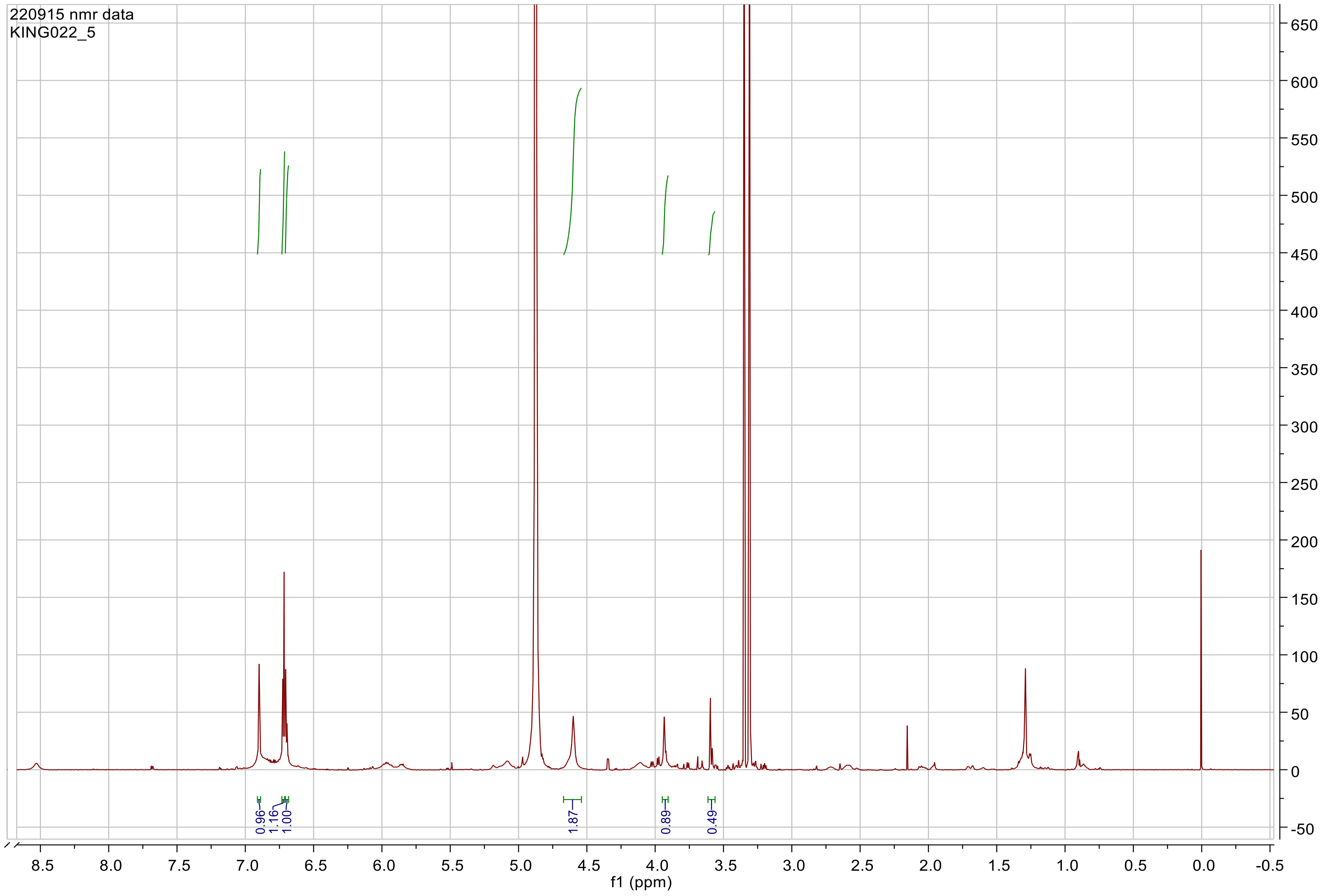

**Figure S5.** The LC-UV chromatogram at 254 nm, UV spectrum and negative-ion mode MS data of **3**

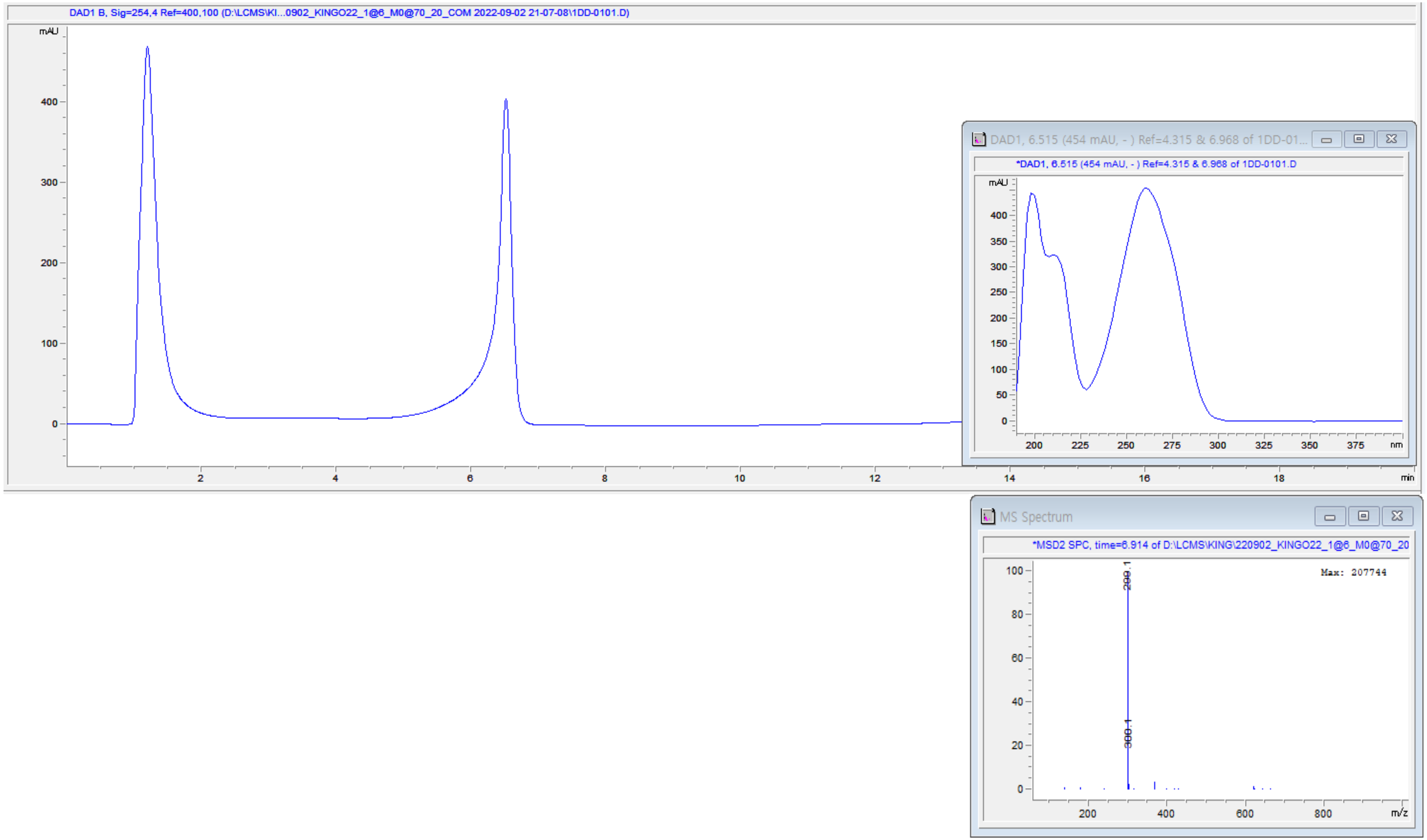

**Figure S6.** The $^{1}$H NMR spectrum of **3** (DMSO, 850 MHz)

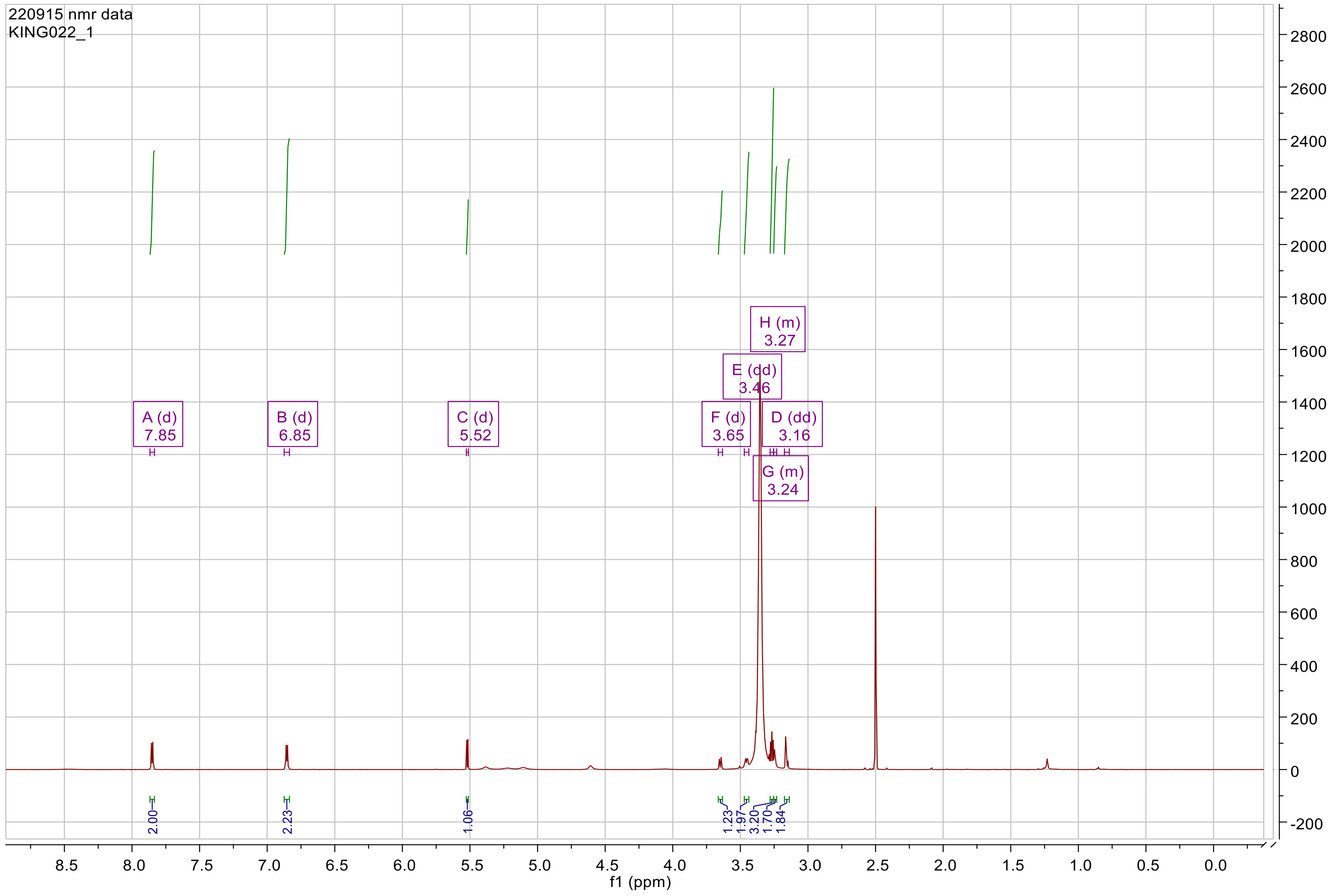

**Figure S7.** The LC-UV chromatogram at 210 nm, UV spectrum and negative-ion mode MS data of **4**

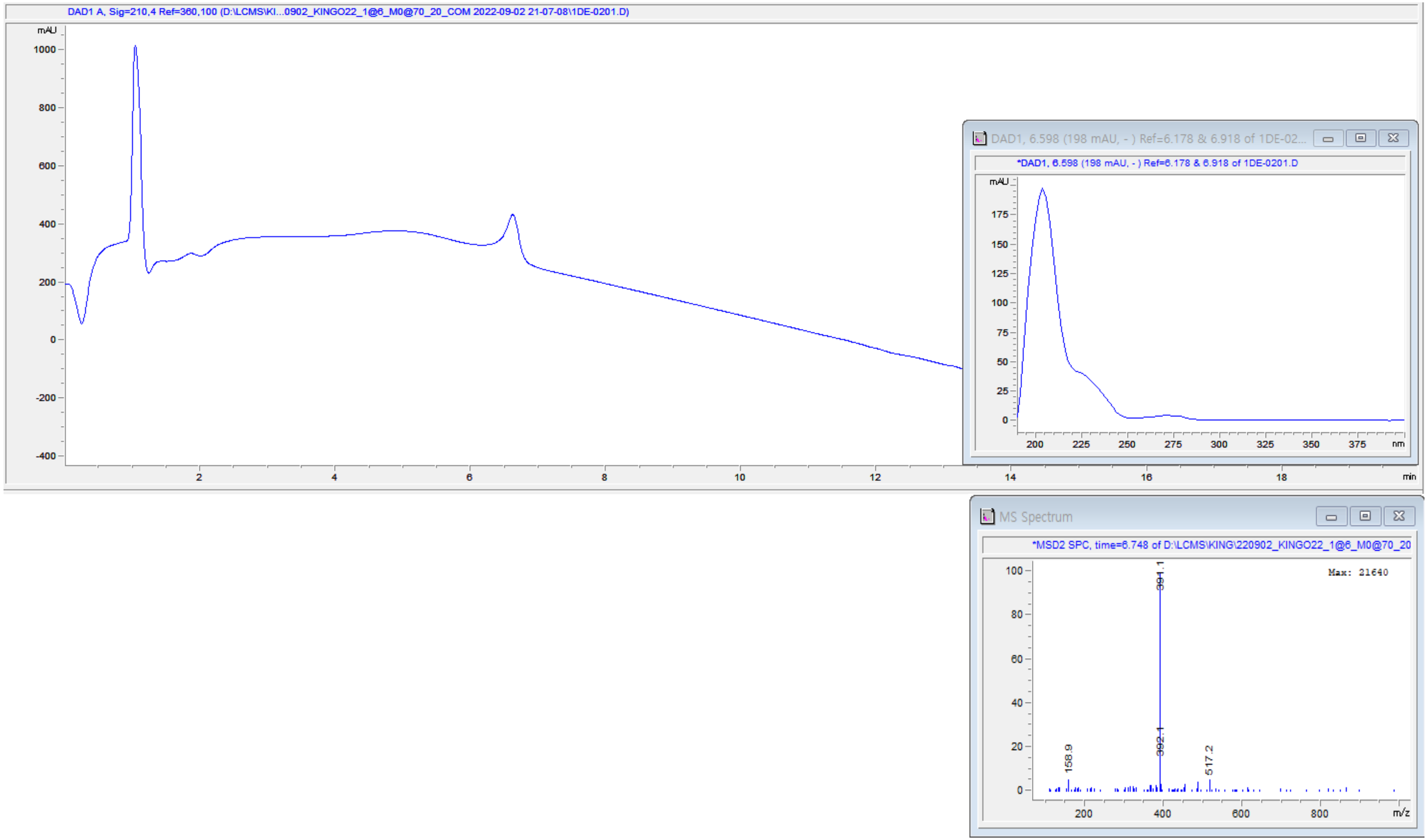

**Figure S8.** The $^{1}$H NMR spectrum of **4** (DMSO, 850 MHz)

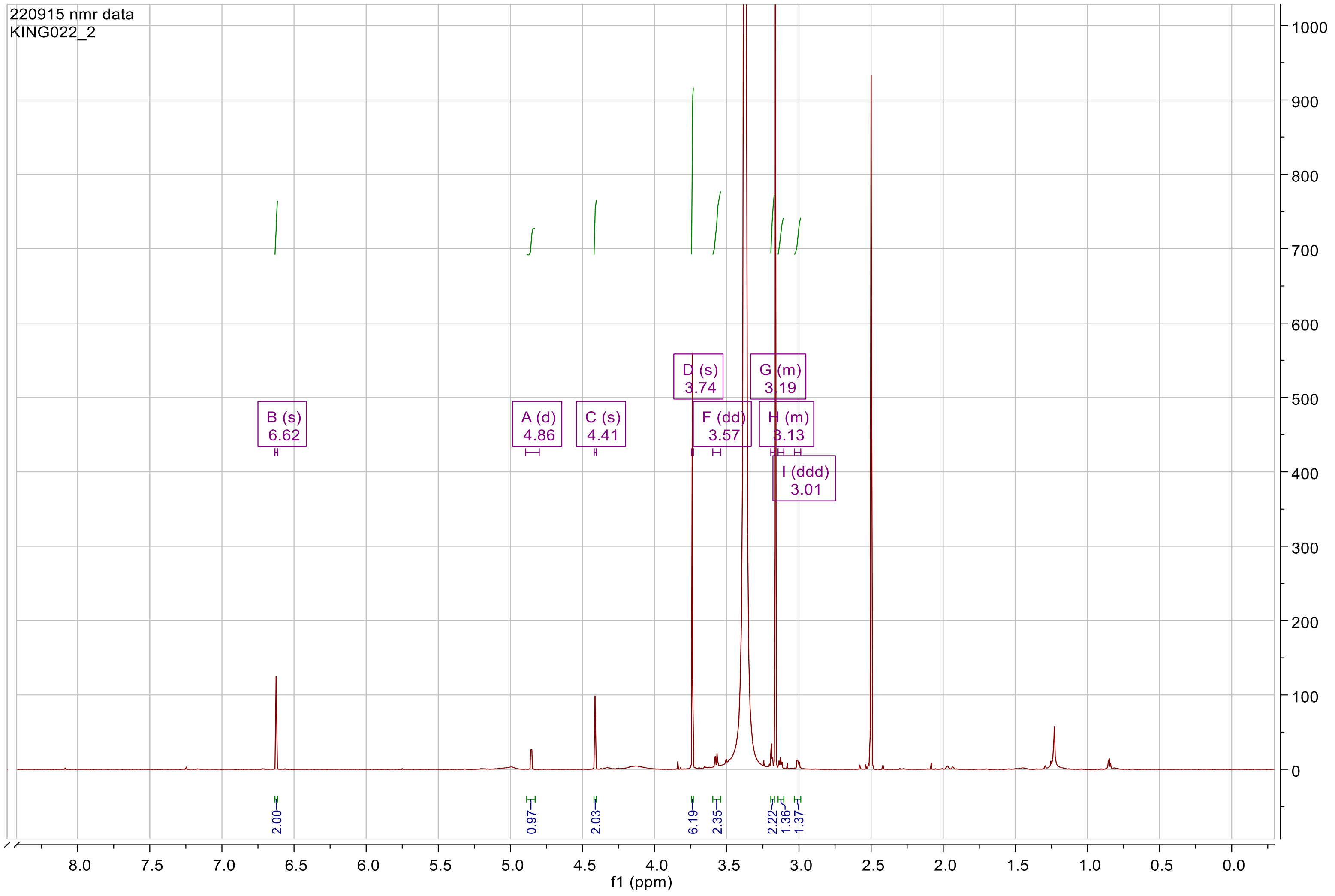

**Figure S9.** The LC-UV chromatogram at 210 nm, UV spectrum and negative-ion mode MS data of **5**

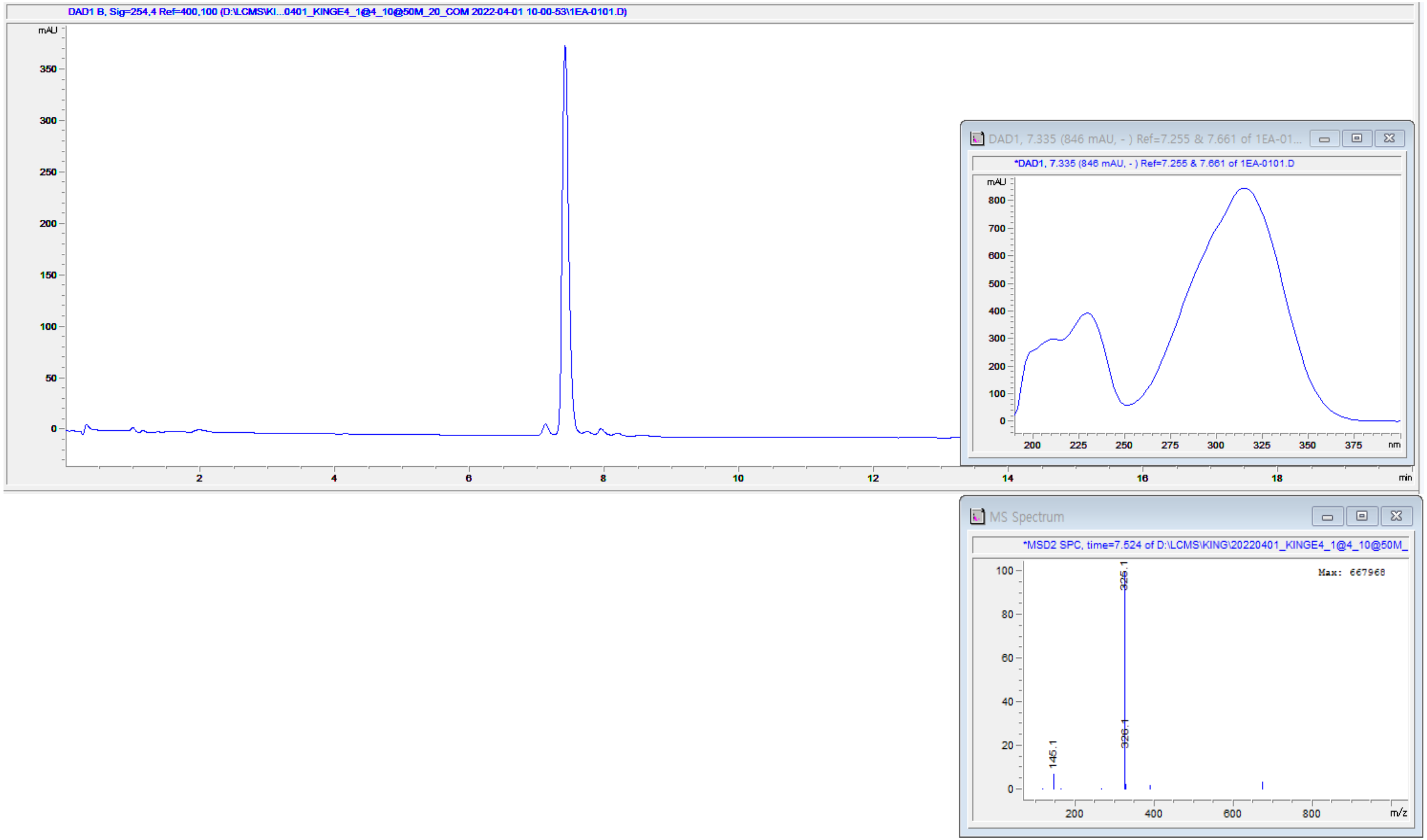

**Figure S10.** The $^1$H NMR spectrum of **5** ($CD_3OD$, 850 MHz)

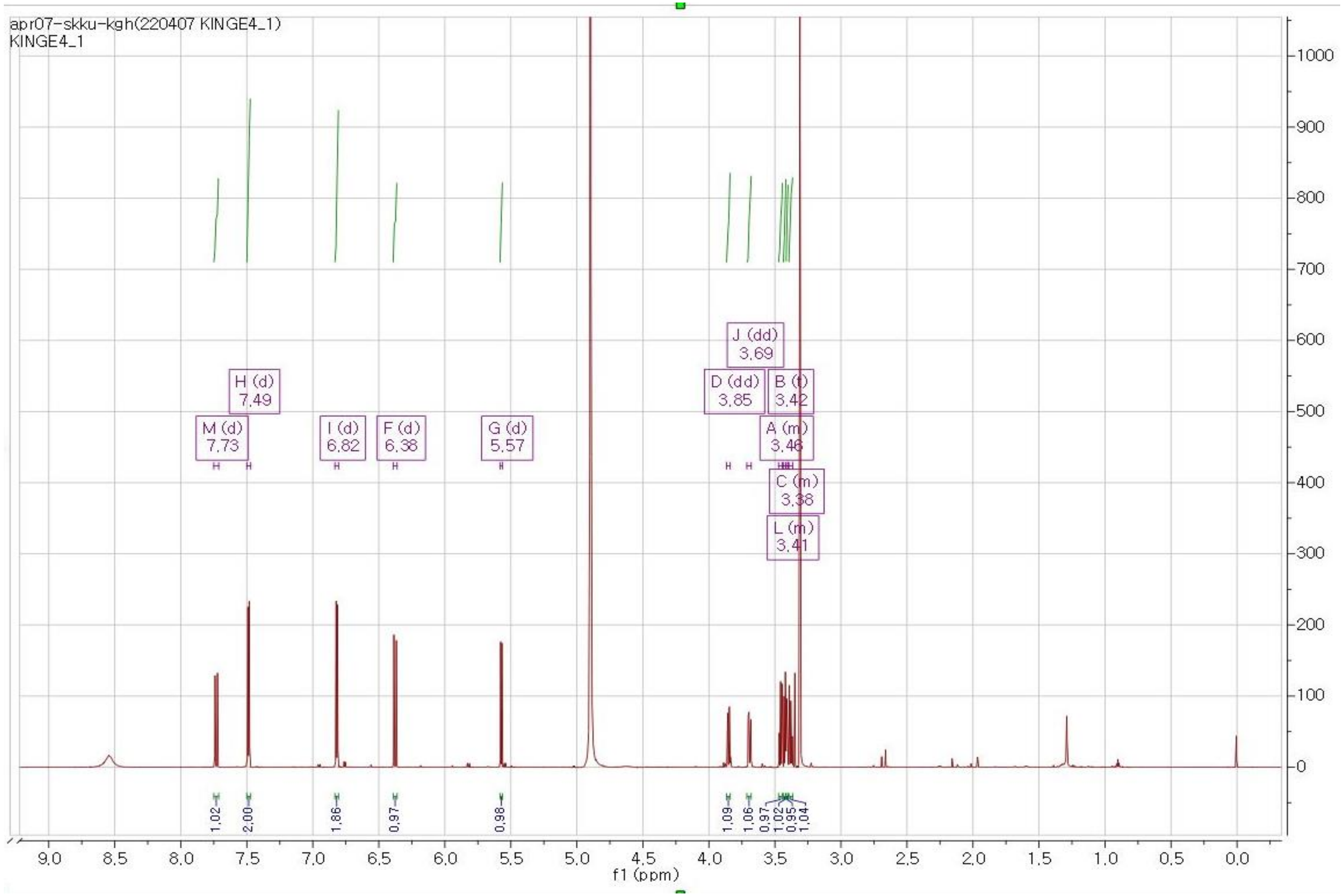

**Figure S11.** The LC-UV chromatogram at 210 nm, UV spectrum and negative-ion mode MS data of **6**

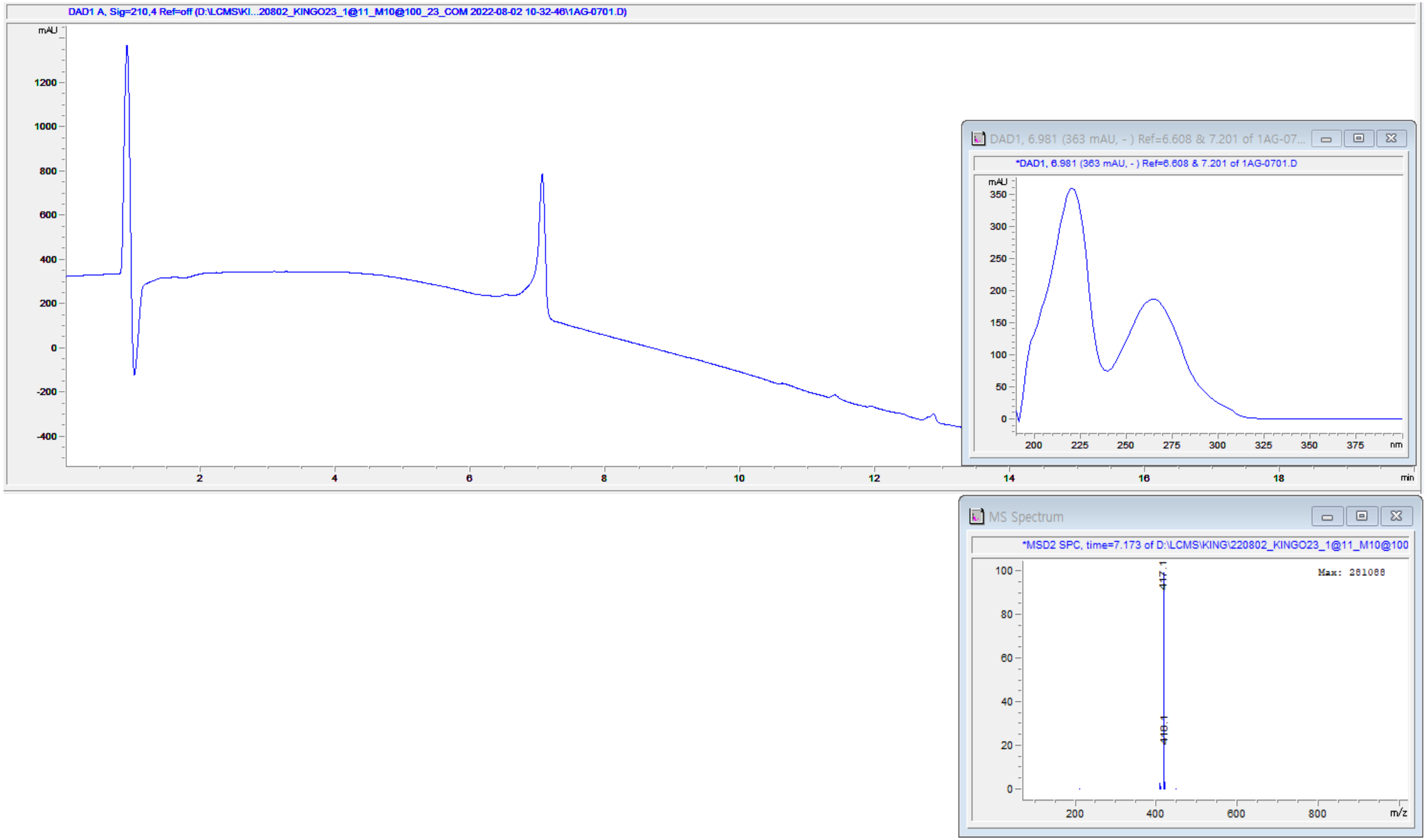

**Figure S12.** The $^{1}$H NMR spectrum of **6** ($CD_3OD$, 850 MHz)

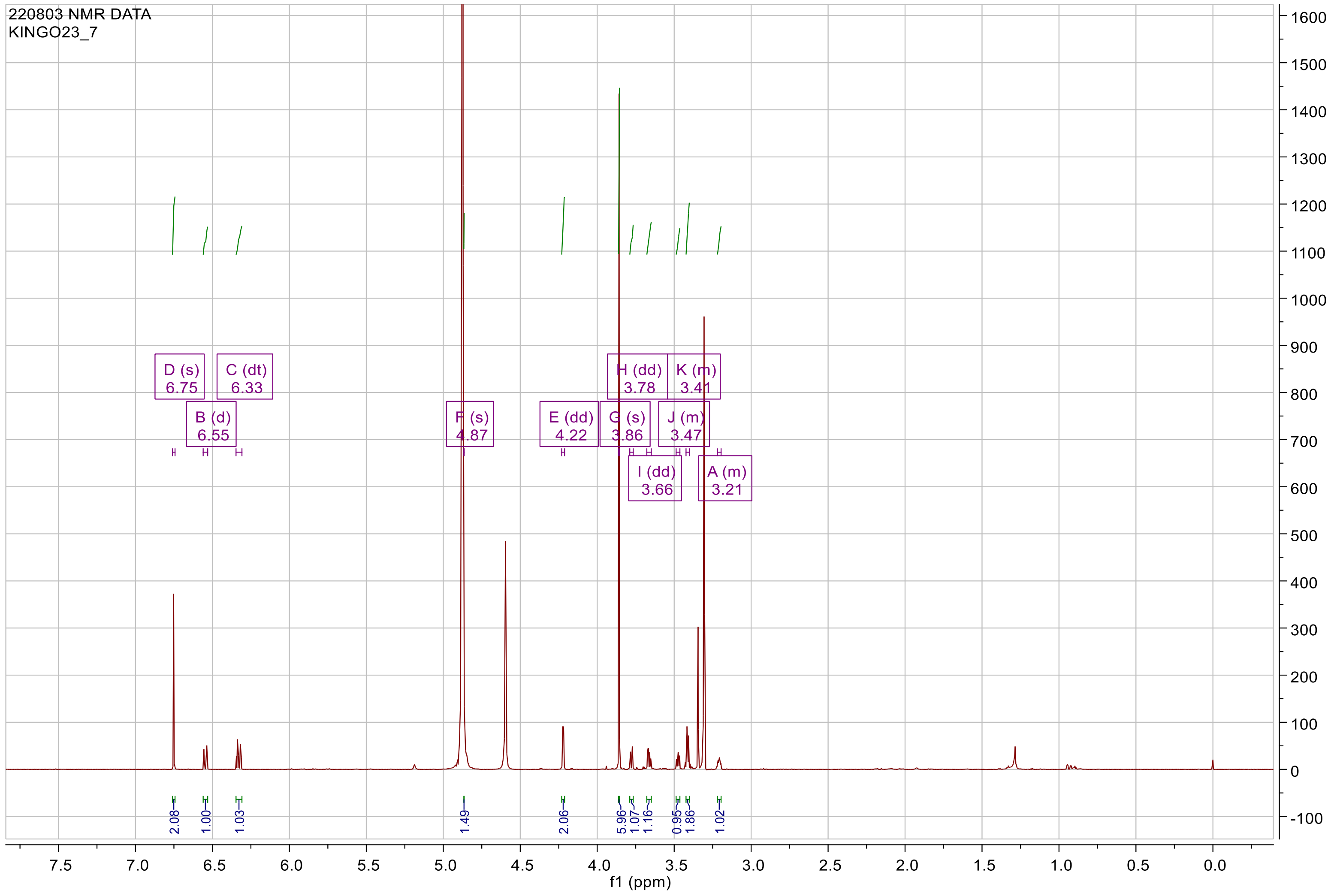

Supplement: Supplementary file 1 [file nutrients-16-01036-s001.zip › nutrients-2861030-supplementary.pdf]
